# Supplementary material for: Patient-physician communication about financial problems: A cross-sectional study among over-indebted individuals
Source: PLoS One. 2020 May 5;15(5):e0232716. doi: 10.1371/journal.pone.0232716 (PMC7199951; doi:10.1371/journal.pone.0232716)
Supplement: Attachment — Submitted filename: Response to Reviewers.pdf [file pone.0232716.s001.pdf]

# Response letter

PONE-D-19-33728
PATIENT-PHYSICIAN COMMUNICATION ABOUT FINANCIAL PROBLEMS: A CROSS-SECTIONAL STUDY AMONG OVER-INDEBTED INDIVIDUALS
PLOS ONE

**Journal Requirements**

When submitting your revision, we need you to address these additional requirements.

1) Please ensure that your manuscript meets PLOS ONE's style requirements, including those for file naming. The PLOS ONE style templates can be found at

http://www.journals.plos.org/plosone/s/file?id=wjVg/PLOSOne_formatting_sample_main_body.pdf and
http://www.journals.plos.org/plosone/s/file?id=ba62/PLOSOne_formatting_sample_title_authors_affiliations.pdf

Response: We appreciate your remark and updated the manuscript to meet PLOS ONE’s style requirements.

2) We note that you have indicated that data from this study are available upon request. PLOS only allows data to be available upon request if there are legal or ethical restrictions on sharing data publicly. For information on unacceptable data access restrictions, please see http://journals.plos.org/plosone/s/data-availability#loc-unacceptable-data-access-restrictions.

Response: We understand your request and explained the restrictions on sharing data from this study in our revised cover letter in more detail.

In your revised cover letter, please address the following prompts:

a) If there are ethical or legal restrictions on sharing a de-identified data set, please explain them in detail (e.g., data contain potentially identifying or sensitive patient information) and who has imposed them (e.g., an ethics committee). Please also provide contact information for a data access committee, ethics committee, or other institutional body to which data requests may be sent.

b) If there are no restrictions, please upload the minimal anonymized data set necessary to replicate your study findings as either Supporting Information files or to a stable, public repository and provide us with the relevant URLs, DOIs, or accession numbers. Please see http://www.bmj.com/content/340/bmj.c181.long for guidelines on how to de-identify and prepare clinical data for publication. For a list of acceptable repositories, please see http://journals.plos.org/plosone/s/data-availability#loc-recommended-repositories.

We will update your Data Availability statement on your behalf to reflect the information you provide.

3) Please ensure you have thoroughly discussed any potential limitations of this study within the Discussion section.

Response: We appreciate your careful review and have carefully reconsidered any potential limitations of this study. As addressed in our cover letter, we would like to confirm that we have described relevant limitations in the original manuscript. We discussed any potential limitations owing to the methods of data collection, limitations of the available data and generalizability of findings. Following your comment and the reviewers' feedback, we also pointed out potential selection bias with regards to eligibility criteria for participants that are required to have sufficient language skills to complete the written questionnaire. We summarized these key aspects in the Discussion section as follows (page 14, lines 313-341):

> "This study has several limitations. First, data on patient-physician communication about financial problems was self-reported. Thus, data might be subject to recall or social desirability bias and differ from actual behaviour. Second, those who visit a debt advice agency to seek help might be less likely to seek additional advice from their GP. Likewise, individuals who lack sufficient language skills to complete the questionnaire were not included in the present study but are likely to face barriers to communication with their GP. Therefore, the prevalence of patient-physician communication in the over-indebted population could be underestimated. Individuals who disclosed communicating about their financial situation with their general practitioner might have been more likely to participate in the study to communicate about their debt-related health problems. Moreover, only patients who had reported having a regular general practitioner were subsequently asked about their communication with their GP. Due to the latter aspects, the prevalence of conversations about financial distress by the over-indebted in the primary care setting might be overestimated. However, this selection bias can be assumed to have a minor effect on results of multiple regression analysis.
>
> Third, on the basis of the available data, it is not possible to identify reasons why patients or general practitioners chose (not) to discuss financial problems but previous studies have addressed this issue [35, 38, 58]. Prior studies identified various strategies used during consultation to deal with health-related expenses in general practice [29, 62]. It remains to be established to what extent the conversations examined in the present study reflect an effective pathway for over-indebted patients and their general practitioners to enhance health outcomes and course of treatment, or to address the overall causes and consequences of financial problems.
>
> Several methodological limitations and country-specific legal consequences of over-indebtedness limit the generalizability of our findings. Nevertheless, the present study reveals a need to raise awareness of patients' financial problems among relevant stakeholders throughout Germany. These findings may also apply to similar health and legal systems because any over-indebted individual can be considered at increased risk of illness and may experience limited access to health care."

## Reviewers' comments

### Reviewer #1

Introduction should be summarized.
Please adhere to STROBE guidelines for improving the quality of reporting of your study.
This could be found at the link of below.
https://strobe-statement.org/index.php?id=available-checklists

Response: We agree with the reviewer's assessment. Accordingly, we have revised the Introduction section to limit the explanation of the scientific background to fewer key findings (page 4, lines 82-94). We further modified the Introduction (page 3f., lines 67-72) by presenting more detailed aspects of co-payments for health services in Germany in the Methods section only. In the Methods section, these details provide key information on cost-sharing that might induce cost-related non-adherence (page 7, lines 173-182):

> "In Germany, adults enrolled in statutory health insurance need to pay co-payments for health services (German Social Code Book V § 61). For instance, co-payments for in-patient care amount to ten euros per calendar day, ten percent of costs for each prescribed medication (min. five, max. ten euros), and an additional fee of ten euros per prescription of therapies such as physiotherapy, speech therapy or occupational therapy [24]. Therefore, Additionally, data on self-reported strategies to cope with health-related expenses were collected, including cost-related medication non-adherence and cutting on necessities to pay for medications (yes; no) in the previous 12 months was assessed. More specifically, the questionnaire captured CRN behaviours such as delaying or not filling prescriptions, skipping or decreasing doses of prescribed medications for financial reasons (yes; no)."

Discussion: it is suggested to discuss a link between communication problems and social determinants of health in discussion section and explain what your study adds to the body of literature in this important field.
Also please recommend directions for future researches on the topic including qualitative studies to deeply understand the causes of not discussing financial costs of medical services either by patients or by physicians.

Response: We thank the reviewer for the thoughtful comment, and have modified the discussion section accordingly. The revised text reads as follows on page 14f. (lines 291-311):

> "An encouraging finding is that some over-indebted patient groups that possibly bear a particularly heavy burden regarding their health status and financial distress are more likely to communicate about their financial problems with their general practitioner. The predictors of patient-physician communication in general practice identified in the present study were in line with several prior patient surveys [35, 40, 41]. However, few studies have examined the link between social determinants and patient-physician communication about financial problems in clinical encounters yet, most of which assessed communication in diverse US patient groups [33–43]. This is the first explorative study of such communication in a population of over-indebted individuals in Germany." The findings highlight that communication about such concerns in clinical encounters is rare even among over-indebted patients who face an increased risk of disease and are particularly susceptible to cost problems with regards to co-payments required for health services in Germany. These results warrant further research to draw conclusions about underlying reasons for differences in communication about financial problems within the over-indebted population. More specifically, qualitative data may help to gain a better understanding of barriers to communication both for patients and physicians.

Please check the manuscript for some grammatical and writing errors. For example, page3 line 72; nonadherence

Response: We have checked the manuscript for errors as you kindly suggested, and replaced the spelling “nonadherence” by “non-adherence” to use British English spelling consistently.

**Reviewer #2**

This study introduced an important issue focused on social economic status and aimed to investigate patient-physician communication about financial problems in general practice among over-indebted individuals. The study conducted among 598 respondents (50.2% response rate) enrolled in statutory health insurance. The prevalence of patient-physician communication about financial problems was 22.6. The author reported that individuals with a high educational level were less likely to report such conversations than those with medium educational level. I think missing to report some of main items make study difficult to understand that I introduced below:

General comments

-Non-response analysis is not provided. How did the selection bias assessed? This should be assessed and discussed in the manuscript.

Response: We thank the reviewer for pointing this out. We have discussed the limitations of the available data on patient-physician communication in the discussion section to clarify potential selection bias in more detail. Due to the following procedure, we assumed selection bias to have minor effects on the results of the present study:

First, a large number of debt advice agencies throughout the federal state of North Rhine-Westphalia, Germany, served as recruiting offices to ensure that a representative sample of over-indebted individuals is included in the present study. In total, 70 of 145 non-profit debt advice agencies agreed to participate. These were all associated with the local German Consumer Organisation or one of the member organisations of the ‘Expert Committee Debt Counselling of Non-statutory Welfare NRW’ (German: Fachausschuss Schuldnerberatung der Freien Wohlfahrtspflege NRW) that invited the agencies to conduct recruitment. Reasons for not participating that were assessed systematically, were mainly temporary time and resource constraints of debt advice agencies. However, the characteristics of the workforce, location and services offered by debt advice agencies were similar for participating and non-participating agencies. Therefore, we assume that this does not affect the study results, and had thus not included this aspect in the original manuscript.

In the revised manuscript, we described this aspect in more detail as follows (Methods section, page 5, lines 109-116):

> “Between July and October 2017, 70 of 145 approved debt advice agencies throughout the German federal state of North Rhine-Westphalia (NRW) conducted the recruitment of participants. Debt advice agencies that provide debt and insolvency counselling services to over-indebted consumers in Germany (Insolvency Statute; German: Insolvenzordnung; §305) were invited to act as recruiters by their umbrella organisation, namely the local German Consumer Organisation or the ‘Expert Committee Debt Counselling of Non-statutory Welfare NRW’ (German: Fachausschuss Schuldnerberatung der Freien Wohlfahrtspflege NRW) [45].”

Second, a high response rate (50.2%) was achieved. Nevertheless, selective non-response might distort the results of the study which we addressed in the Discussion section (page 14f., lines 315-327):

> "[…] those who visit a debt advice agency to seek help might be less likely to seek additional advice from their GP. Likewise, individuals who lack sufficient language skills to complete the questionnaire were not included in the present study but are likely to face barriers to communication with their GP. Therefore, the prevalence of patient-physician communication in the over-indebted population could be underestimated. Individuals who disclosed communicating about their financial situation with their general practitioner might have been more likely to participate in the study to communicate about their debt-related health problems. Moreover, only patients who had reported having a regular general practitioner were subsequently asked about their communication with their GP. Due to the latter aspects, the prevalence of conversations about financial distress by the over-indebted in the primary care setting might be overestimated. However, this selection bias can be assumed to have a minor effect on results of multiple regression analysis."

Finally, all participants were informed about the anonymity of the survey which also contributed to minimizing non-response bias.

-sample size calculation was missed.

Response: We appreciate the Reviewer's careful review. As the present study was an explorative study, a key target was to achieve a high response rate to ensure a representative and non-selective sample. We estimated a sample size a priori based on a previous study of an over-indebted population in Germany [Ochsmann EB, Rueger H, Letzel S, Drexler H, Münster E. Over-indebtedness and its association with the prevalence of back pain. BMC Public Health. 2009;9:451. doi:10.1186/1471-2458-9-451]. Each debt advice agency received a specific amount of study material according to the number of clients and advisors at each site (mean 24; min. 5 to max. 100 questionnaires). This procedure was determined a priori to ensure that standardised data collection was feasible for debt advisors involved in recruitment. Subsequently, each advisor handed out the study material to clients when these were identified eligible during consultations between July and October 2019.

We have addressed the sampling procedure in the Methods section in more detail to clarify this aspect (page 5f., lines 112-126):

> "Debt advice agencies that provide debt and insolvency counselling services to over-indebted consumers in Germany (Insolvency Statute; German: Insolvenzordnung; §305) were invited to act as recruiters by their umbrella organisation, namely the local German Consumer Organisation or the 'Expert Committee Debt Counselling of Non-statutory Welfare NRW' (German: Fachausschuss Schuldnerberatung der Freien Wohlfahrtspflege NRW) [45]. Each debt advice agency that agreed to participate received a specific amount of study material that corresponded to the number of clients and advisors at each site identified a priori (mean 24; min. 5 to max. 100 questionnaires). Data was collected by a self-administered written questionnaire returned to the study centre by mail: Clients received an anonymous standardised questionnaire and a postage-paid return envelope from their counsellor after the consultation when these were identified eligible according to the following criteria: a) completed at least an initial consultation based on the premise that it reflects a

> sensitive situation necessary to build trust; b) minimum age of 16 years due to contractual capability; c) sufficient language, reading and writing skills owing to the data collection method; d) one participant per household.”

-please elaborate how data gathering was done? as study reported that it was self-reported.

Response: We appreciate the reviewer’s suggestion and have revised the Methods section (page 5f., lines 117-126) to clarify the process of data collection:

> “Each debt advice agency that agreed to participate received a specific amount of study material that corresponded to the number of clients and advisors at each site identified a priori (mean 24; min. 5 to max. 100 questionnaires). Data was collected by a self-administered written questionnaire returned to the study centre by mail: Clients received an anonymous standardised questionnaire and a postage-paid return envelope from their counsellor after the consultation when these were identified eligible according to the following criteria: a) completed at least an initial consultation based on the premise that it reflects a sensitive situation necessary to build trust; b) minimum age of 16 years due to contractual capability; c) sufficient language, reading and writing skills owing to the data collection method; d) one participant per household.”

-why 70 advice agencies was selected? Is that all or selected how they were selected?

Response: We thank the reviewer for the helpful comment and revised the manuscript to clarify the selection of debt advice agencies. As pointed out above, all approved non-profit debt advice agencies throughout the federal state of North Rhine Westphalia, Germany were invited to act as recruiters: The local German Consumer Organisation and the ‘Expert Committee Debt Counselling of Non-statutory Welfare NRW’ (German: Fachausschuss Schuldnerberatung der Freien Wohlfahrtspflege NRW) invited the agencies to conduct recruitment. In total, 70 of 145 debt advice agencies that were all associated with these organisations agreed to participate. During the data collection process, we systematically assessed reasons of debt advice agencies not to participate. These were mainly temporary time and resource constraints of the agencies. However, the characteristics of the workforce, location and services offered by debt advice agencies were similar for participating and non-participating agencies which is why we did not elaborate on the latter aspect in the manuscript in more detail.

The revised text reads as follows (Methods section, page 5, lines 109-116):

> “Between July and October 2017, 70 of 145 approved debt advice agencies throughout the German federal state of North Rhine-Westphalia (NRW) conducted the recruitment of participants. Debt advice agencies that provide debt and insolvency counselling services to over-indebted consumers in Germany (Insolvency Statute; German: Insolvenzordnung; §305) were invited to act as recruiters by their umbrella organisation, namely the local German Consumer Organisation or the ‘Expert Committee Debt Counselling of Non-statutory Welfare NRW’ (German: Fachausschuss Schuldnerberatung der Freien Wohlfahrtspflege NRW) [45].”

-how Clients were recruited to the study?

Response: Following the reviewer's constructive comments on the recruitment process and data collection (see above), we have revised the respective paragraph as follows for clarification (Methods section, page 5, lines 117-126):

> "Each debt advice agency that agreed to participate received a specific amount of study material that corresponded to the number of clients and advisors at each site identified a priori (mean 24; min. 5 to max. 100 questionnaires). Data was collected by a self-administered written questionnaire returned to the study centre by mail: Clients received an anonymous standardised questionnaire and a postage-paid return envelope from their counsellor after the consultation when these were identified eligible according to the following criteria: a) completed at least an initial consultation based on the premise that it reflects a sensitive situation necessary to build trust; b) minimum age of 16 years due to contractual capability; c) sufficient language, reading and writing skills owing to the data collection method; d) one participant per household."

-title of table 1 should be re-write and revised, it was illegible.

Response: Following the reviewer's comment, we corrected this error in the manuscript.

-Some of variables like educational level, financial distress, chronic diseases, and etc. were unclear in measurements.

Response: We agree with the reviewer that it might be useful to provide further details on the measurement of variables. Therefore, we have revised the Methods section (page 6ff., lines 133-182) for clarification:

> "Sociodemographic information included sex, age, educational level, employment status, migrant background, marital status and number of children to account for general differences in patient-physician communication patterns [46–53]. We classified age into three age groups (18-29; 30-49; 50-79 years). Self-reported data on the highest general educational and vocational qualifications were classified into three levels of education using the International Standard Classification of Education (ISCED) [54]: We distinguished between low (primary education, lower secondary education), medium (upper secondary education, post-secondary non-tertiary education) and high educational levels (tertiary education). Participants that reported full, part-time or marginal employment were classified as employed. In our study, we assumed that those with a lower SES (low educational level; unemployment), on the one hand, might feel compelled to discuss financial problems with their GP or have limited communication abilities that prevent such dialogue, on the other hand. Those with a higher SES might be especially reluctant to disclose financial problems due to feelings of shame, and may adopt different coping strategies. A migrant background was assumed when participants or at least one parent were born outside of Germany. Factors such as language barriers or differences in beliefs about illness and patient-physician interaction may hinder communication about financial problems in those with a migrant background [48, 53]. We classified participants' marital status into three groups: married, previously married (divorced or widowed), and never married. Number of children was classified into three groups (no children; 1 child; 2 or more children). The latter two variables were taken into account to examine patients' social support linked to marital status and household living expenses that vary by the number

of children, and may influence communicative behaviour and interaction with their GP.

Patients' needs and expectations that can influence patient-physician communication might also depend on health status, disease stage and course of treatment [50]. Therefore, we considered both chronic diseases and recent visit to a general practitioner in the statistical analysis. Participants self-reported any chronic health conditions (yes, - please specify; no; don't know) and medication use in the last seven days. Medical experts reviewed self-reported data on both chronic conditions and medication use (pharmaceutical, underlying health condition) to identify and categorize chronic diseases according to ICD-10-GM (German adaptation of the International Statistical Classification of Diseases and Related Health Problems). Participants reported the use of outpatient and inpatient health care in the previous 12 months, including a visit to the GP (yes; no).

Moreover, we assumed that a high degree of financial distress increases individuals' perceived need for communication about financial problems. Thus, we included the following measures in the analysis to account for the degree of stress related to debt and cost of illness as well as patients' strategies to cope with their financial problems: The level of self-reported subjective financial distress due to debt was measured on a five-point Likert-scale which was dichotomized to distinguish low (not at all; somewhat; moderately) and high financial distress (to a great extent; to a very great extent). In Germany, adults enrolled in statutory health insurance need to pay co-payments for health services (German Social Code Book V § 61). For instance, co-payments for in-patient care amount to ten euros per calendar day, ten percent of costs for each prescribed medication (min. five, max. ten euros), and an additional fee of ten euros per prescription of therapies such as physiotherapy, speech therapy or occupational therapy [24]. Therefore, data on self-reported strategies to cope with health-related expenses were collected, including cost-related medication non-adherence and cutting on necessities to pay for medications (yes; no) in the previous 12 months was assessed. More specifically, the questionnaire captured CRN behaviours such as delaying or not filling prescriptions, skipping or decreasing doses of prescribed medications for financial reasons (yes; no)."

-Each table should be inserted in one page. Tables 1 and 2 were placed in one page and half of table 2 is placed in next page.

Response: We appreciate the reviewer's careful assessment and have reviewed the formatting rules required by PLOS ONE. In order to comply with these instructions, we have placed each table in our manuscript directly after the paragraph in which it is first cited.

-how participant were estimated over indebted?

Response: We appreciate the reviewer's query about the definition of over-indebted participants and have revised the Methods section (page 8, lines 107-116) to specify that we considered all clients across the selected debt advice agencies as "over-indebted" because these are the target group of such counselling agencies throughout Germany:

"This cross-sectional survey among clients of debt advice agencies examined health, medication use and self-medication in the over-indebted (OID survey; German acronym: ArSemü) [44]. Between July and October 2017, 70 of 145 approved debt

advice agencies throughout the German federal state of North Rhine-Westphalia (NRW) conducted the recruitment of participants. Debt advice agencies that provide debt and insolvency counselling services to over-indebted consumers in Germany (Insolvency Statute; German: Insolvenzordnung; §305) were invited to act as recruiters by their umbrella organisation, namely the local German Consumer Organisation or the ‘Expert Committee Debt Counselling of Non-statutory Welfare NRW’ (German: Fachausschuss Schuldnerberatung der Freien Wohlfahrtspflege NRW) [45].”
